# Moving polewards in winter: a recent change in the migratory strategy of a pelagic seabird?

**DOI:** 10.1186/1742-9994-7-15

**Published:** 2010-05-19

**Authors:** Petra Quillfeldt, Juan F Masello, Rona AR McGill, Mark Adams, Robert W Furness

**Affiliations:** 1 Max-Planck-Institut für Ornithologie, Vogelwarte Radolfzell, Schlossallee 2, 78315 Radolfzell, Germany; 2 NERC Life Sciences Mass Spectrometry Facility, Scottish Universities Environmental Research Centre, East Kilbride, Glasgow G75 OQF, UK; 3 Dept. of Zoology, The Natural History Museum, Akeman Street, Tring, Herts, HP23 6AP, UK; 4 Faculty of Biomedical and Life Sciences, University of Glasgow, Glasgow G12 8QQ UK

## Abstract

**Background:**

During the non-breeding period, many birds migrate to milder areas, found closer to the equator than their breeding sites. Opposite movements are very rare. In the Southern Ocean, the abundance of ^13^C declines markedly with more southern latitude, providing a characteristic ^13^C isoscape. This can be used as a tracer for the movement of seabirds between breeding and inter-breeding areas, by comparing stable isotope ratios of feathers grown at different times of the year.

**Results:**

We studied seasonal movements of Thin-billed prions (Aves, Procellariiformes), breeding at the Subantarctic Falkland/Malvinas Islands, compared with those of Wilson's storm-petrels breeding in the Antarctic South Shetland Islands. The two species showed opposite migratory movements. While Wilson's storm-petrels moved to warmer waters north of the Drake Passage in winter, Thin-billed prions showed a reversed movement towards more polar waters. Carbon stable isotope ratios in recent and historical feathers indicated that poleward winter movements of Thin-billed prions were less common historically (45% in 1913-1915), and have only recently become dominant (92% in 2003-2005), apparently in response to warming sea temperatures.

**Conclusions:**

This study shows that pelagic seabirds can rapidly change migration strategies within populations, including migration towards more poleward waters in winter.

## Background

Migration is thought to be an adaptive strategy, when resources found in the breeding area during the breeding season become scarce during the non-breeding season or climate becomes unfavourable. Animals can then displace to more productive or milder areas [1]. The places animals select for the winter season are usually warmer, e.g. transequatorial to winter in the opposing hemisphere's summer, or further towards the equator within the same hemisphere (latitudinal migration) or lower in the mountains (altitudinal migration). Opposite movements are very rare, and virtually absent in terrestrial birds [1].

Many seabirds are too small to carry devices presently available for satellite or GPS tracking. Small pelagic seabirds are difficult to observe in their vast marine ecosystems, and little is known about their distribution and behaviour outside the breeding season. Stable isotopes provide a powerful tool to study movements and trophic position of such birds if tissue grown at different times during the year can be matched to an isotopic gradient across the area of movement [2].

Since keratin is a highly stable molecule, the nitrogen and carbon isotopic composition of feathers remains unchanged after the completion of growth. Thus, historical feathers provide a window that allows us to look back in time, and stable isotope analysis can therefore be applied to study changes in migratory patterns due to environmental change. Historical feathers from museum specimens have been analysed for stable isotopes in few seabird species, chiefly to investigate reasons for population change. Declining δ^15^N levels, indicative of declining trophic levels, were found in Northern Fulmars *Fulmarus glacialis *[3], and Marbled Murrelets [4,5]. Declining carbon isotope ratios were found in Rockhopper penguins *Eudyptes chrysocome*, and were interpreted as a decline in primary productivity and thus, the carrying capacity, of the ecosystem [6,7]. The aim of the present study is to use stable isotopes to understand the movements of a small pelagic seabird, both from recent and historical samples.

Previous studies suggested that the carbon stable isotope ratio δ^13^C in the Southern Ocean declines between 40 and 80°S [8-11]. By compiling data from the literature and modeling this trend, we here aim to verify if seasonal latitudinal movements of seabirds in the Southern Ocean will result in different δ^13^C values of adult feathers grown during winter, and chick feathers and induced adult feathers grown during the summer. The latter both represent the breeding season equally well and do not differ in their stable isotope ratios (see Methods).

**Figure 1:**
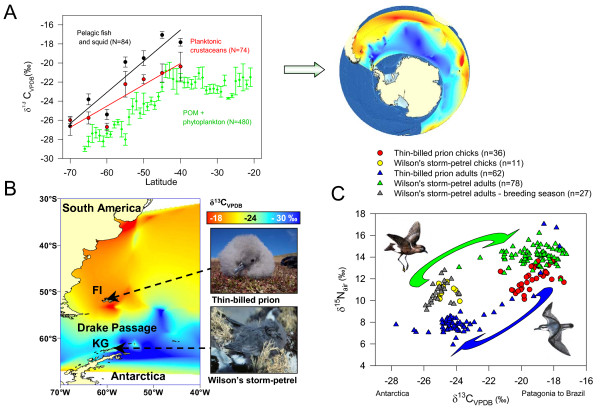
**Carbon stable isotope ratios and breeding sites**. **A**. Carbon stable isotope ratios in Southern Ocean plankton and predators (fish and squid), The sample size refer to discrete species × location data points. An interpolated isoscape was derived from phytoplankton data. Data points are from a review of available literature. **B**. Breeding sites of the two study species (KG = King George Island, South Shetlands, FI = New Island, Falkland Islands/Malvinas), distribution of isotope values from the interpolated isoscape in the study ares, and pictures of corresponding chicks. **C**. Stable isotope ratios of feathers representing diet during the breeding season (chick feathers and induced adult feathers) and naturally moulted adult feathers (representing the interbreeding season), showing opposite movement of Thin-billed prions and Wilson's storm-petrels during migration

We then applied the results of the spatial model to interpret data on seabird movements. Our focal species in this study was an abundant pelagic seabird in the Southern Ocean, the Thin-billed prion *Pachyptila belcheri*, sampled at a breeding site at New Island Nature Reserve in the Falkland/Malvinas Islands, at 52°S (Fig. 1B). Thin-billed prions breed on Sub-Antarctic islands. Their year-round distribution is poorly known. They are highly pelagic and mobile, scatter widely and are not attracted by ships, making their observation in Southern Ocean winter waters difficult. Based on stable isotopes of adult feathers grown during winter, it has been suggested recently that Thin-billed prions move towards more polar waters for the winter, both from their breeding populations from Kerguelen [12,13] and the Falkland Islands [10].

We included samples of Wilson's storm-petrels *Oceanites oceanicus *from a breeding site at the South Shetland Islands at 62°S (Fig. 1B), as a reference for the location (δ^13^C) and relative trophic level (δ^15^N). Wilson's storm-petrels feed in Antarctic waters during the chick-rearing period, and are known to migrate north in winter [9,14].

To compare historical and recent migration patterns, we obtained feather samples of Thin-billed prions from skin collections, including only birds collected during the breeding season in the Falkland Islands or adjacent waters. The period 1913 - 1915, ninety years before the present study, was particularly well represented in museums. We compared the historical and recent samples after correcting for long-term-changes in δ^13^C due to the Suess effect (see methods below).

In summary, the aim of the present analysis was to model the δ^13^C distribution in the Southern Ocean as baseline for migration studies and to compare the migration pattern of Thin-billed prions in recent and historic times.

## Results

### Southern ocean isoscape

We compiled data from the literature to model the δ ^13^C distribution (Fig. 1A). Because ^13^C was more enriched in higher trophic levels (Fig. 1A), we only used stable isotope ratios of pelagic primary producers to calculate a δ^13^C isoscape. The Southern Ocean in general, and the Southwest Atlantic in particular, exhibit a strong gradient in carbon stable isotope baseline values (Fig. 1A and 1B, see Fig. S1 in Supplementary material for additional details). This steep gradient ceases abruptly and completely at around 40-45°S, the location (variable in latitudinal position around the globe) of the northern boundary of the Southern Ocean, defined through the Southern Subtropical Front.

### Recent feathers - comparison between two species

Feathers grown during the breeding season reflected the more northerly breeding site of Thin-billed prions at the Falkland Islands, compared to Wilson's storm-petrels, breeding south of the Drake Passage (Fig. 1B,C). δ^13^C varied with species and time of the year, while the largest variation in the data was explained by the interaction between species and time (Table 1). The data thus confirm opposite migration movements: Most Thin-billed prions spent the summer north of the Polar Front, i.e. the northern limit of the Drake Passage, and the winter south, while Wilson's storm-petrels showed the opposite distribution (Fig. 1C). The δ^13^C values for Thin-billed prions in winter were very similar to those of Wilson's storm-petrels in summer (-23.7 ± 0.2‰ vs. -24.9 ± 0.1‰) and vice versa (-19.2 ± 0.1‰ for both Thin-billed prions in summer and Wilson's storm-petrels in winter). Some individuals of both species differed strikingly from the most common isotopic values, demonstrating some intra-specific flexibility in winter distribution.

**Table 1:** Differences between species and times of the year in stable isotope ratios.

Source	Type III Sum of Squares	*df*	Mean Square	*F*	*P*	*η^2^*
Dependent: δ^13^C						
species	17.9	1	17.9	9.7	0.002	0.044
time	16.6	1	16.6	9.0	0.003	0.041
species × time	1226.7	1	1226.7	666.1	<0.001	0.760
Error	386.7	210	1.8			
Total	1698.9	213				
Dependent: δ^15^N						
δ^13^C	138.2	1	138.2	131.9	<0.001	0.387
species	303.1	1	303.1	289.4	<0.001	0.581
time	18.3	1	18.3	17.5	<0.001	0.077
species × time	3.5	1	3. 5	3.3	0.070	0.016
Error	218.9	209	1.0			
Total	1540.3	213				

**Figure 2:**
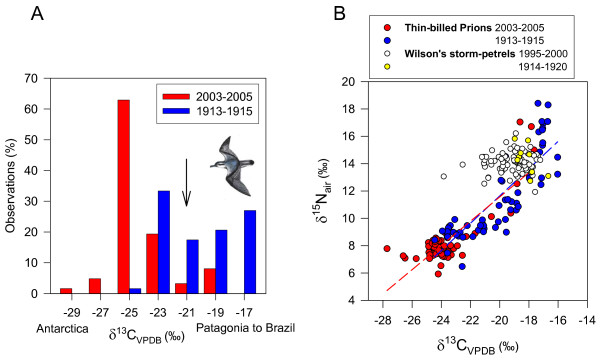
**Differences in carbon stable isotope ratios of feathers of adult Thin-billed prions**. Recent samples (N = 62) were collected at New Island, and historical samples (N = 62) are from museum specimens. Histogram categories in 2A had a width of 2‰, with labels showing the midpoint. In 2B, recent and historic samples of Wilson's storm-petrels were included for comparison.

Carbon and nitrogen stable isotope ratios were measured in feathers of Thin-billed prions and Wilson's storm-petrels (winter vs. summer), tested using GLM. Location (δ^13^C) was included in the model for nitrogen to account for differences in baseline levels with latitude (e.g. see Fig. 2B).

The δ^15^N signatures show that Wilson's storm-petrels maintained a relatively higher trophic level over the year (Fig. 1C). δ^15^N was best explained by species differences (P < 0.001, *η*^2 ^= 0.581), followed by location (represented by δ^13^C values: P < 0.001, *η*^2 ^= 0.387), while time and time*species interactions were of minor importance (Table 1).

### Comparison with historical feathers

Historical and recent feathers of Thin-billed prions were isotopically distinct (Fig. 2A, Wilk's λ = 0.65, P < 0.001). This was due to 3.3‰ lower carbon isotope ratios in recent compared with historical feathers (-23.7 ± 0.2‰ vs. -20.4 ± 0.3‰). Thus, more Thin-billed prions (92%) moult in Antarctic waters now than historically (45%), using a cut-off point of -21‰ (see Fig. 2A). This was based on a frequency distribution of observed δ^13^C values, which had a bimodal distribution, with a minimum at -21 ‰ (Fig. 2A). The nitrogen stable isotope ratio remained constant over time (Table 2: P = 0.51, see also Fig. 2B for similar regression lines).

**Table 2:** Comparison of nitrogen stable isotope ratios of Thin-billed prions breeding in the Falkland Islands.

Source	Type III Sum of Squares	*df*	Mean Square	*F*	*P*	*η^2^*
Dependent: δ^15^N						
δ^13^C	577.4	1	577.4	300.7	<0.001	0.713
time	0.8	1	0.8	0.4	0.510	0.004
Error	232.3	121	1.9			
Total	1078.6	123				

Differences between historical and recent feathers were tested using GLM. Location (δ^13^C) was included in the model to account for differences in baseline levels with latitude (e.g. see Fig. 2B).

**Figure 3:**
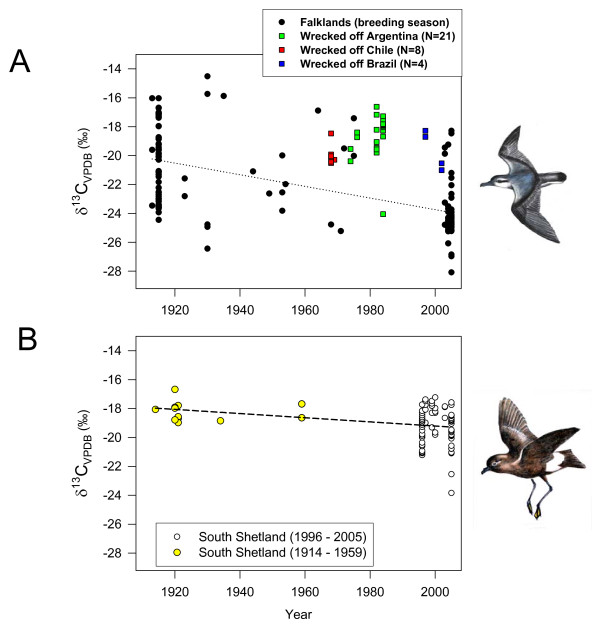
**Stable isotope ratios of Thin-billed prions from wrecks**. Carbon stable isotope ratios of birds found dead on beaches in winter, compared to birds sampled in the Falklands Islands in the breeding season. The dotted trendline was calculated from the breeding season birds only, but the clustered data distribution did not allow us a regression analysis. In 3B, recent and historic samples of Wilson's storm-petrels were included for comparison.

We analysed samples from eight Thin-billed prions collected at Rinconada beach in Chile in the summers 1968 and 1969, 21 birds from Argentinean beaches, collected between 1974 and 1984 and four birds collected at beaches in Brazil in 1997 and 2002 (Fig. 3). All except one bird had carbon isotope ratios indicating northern moulting areas.

In Wilson's storm-petrels, historical and recent feathers were also isotopically distinct (Fig. 2A, Wilk's λ = 0.93, P = 0.043), but the high Wilk's λ (close to 1) and P-value (close to 0.05) indicated that the group means are by far less different than those observed in Thin-billed prions (Fig 2B). Comparatively small differences (1.0‰) were observed in mean carbon isotope ratios in recent compared with historical feathers of Wilson's storm-petrels (-19.2 ± 0.1‰ vs. -18.2 ± 0.2‰; t = 2.5, d.f. 85, P = 0.015), while nitrogen stable isotope ratios did not differ (t = 0.9, d.f. 85, P = 0.368).

## Discussion

In the present study, we found that two small pelagic seabirds had contrasting migratory patterns, and that the recent distribution of moulting Thin-billed prions with predominantly poleward winter migration differed from that observed historically. The trophic level of Thin-billed prions, in contrast, remained constant over time, suggesting that prions responded to changes in their environment by moving to a different location, while any possible changes in diet would be limited to dietary sources of similar trophic level. Wilson's storm-petrel feathers showed a much smaller change in carbon isotope ratios (Fig. 2B and 3B), indicating that baseline level changes alone would not explain the observed differences in Thin-billed prions. In addition, the shift in isotope ratio in Thin-billed prions is really large, almost certainly too large to be caused by changing primary productivity.

Consistency in trophic level is also seen in the comparison between the species, where Wilson's storm-petrels maintained a relatively higher trophic level over the year (Table 1), and in historical feathers (Fig. 2B). This is consistent with observations from regurgitated food during the breeding season. Wilson's storm-petrels commonly took Antarctic krill *Euphausia superba *and lanternfish *Electrona antarctica *of 15-50 mm during summer [15], while Thin-billed prions fed predominantly on small crustaceans of 2-20 mm, mainly amphipods *Themisto gaudichaudii*, copepods *Calanus spec*., krill *Euphausia lucens *and lobster krill *Munida gregaria *[10,16]. Thus, Thin-billed prions, although nearly four times heavier than Wilson's storm-petrels, consistently took smaller prey and fed at a lower trophic level than the storm-petrels, as reflected in lower δ^15^N signatures (Fig. 1C).

The observed differences in the migratory strategies between the species can most likely be explained by differences in the body size and in the abundance and distribution of their preferred prey. Wilson's storm-petrels are the smallest Antarctic endotherms at 38 g, and low winter temperatures might constrain their distribution. In winter, they scatter widely but are often observed over the Patagonian Shelf. Lanternfish (myctophiids) are the most abundant small pelagic fish in the area, with larvae occurring throughout the year [17]. As lanternfish are one of the preferred prey of Wilson's storm-petrels [15], the shelf-break zone offers good feeding opportunities for them in winter. Additionally, Wilson's storm-petrels attend feeding flocks following fisheries vessels to pick up small pieces of discards and may benefit from increased human activity [18].

In contrast, Thin-billed prions do not attend vessels. They depend on lower trophic level prey, especially amphipods *Themisto gaudichaudii *[10,16], which occur throughout the Southern Ocean including the Patagonian shelf [19], mainly feeding on copepods. The ecosystem of the Patagonian shelf is strongly influenced by temperature. Massive blooms of gelatinous zooplankton occurred when the water temperature rose by 2°C, rendering the tidal fronts off Patagonia less prominent and depressing copepod populations [20]. Rising sea temperatures might therefore cause significant bottom-up effects in the food chain.

Could such effects have caused the shift in the frequency of migration routes of Thin-billed prions? High rates of genetic change in preferred migratory direction have been observed in blackcaps *Sylvia atricapilla *as a consequence of assortative mating [21], indicating that the evolution of new migratory preferences can be remarkably rapid in birds. It is not known to which degree the direction of migration is genetically determined in seabirds. A recent study on the faithfulness of individual Thin-billed Prions to a moulting area within and among years suggested a flexible migratory strategy [22]. Feathers of marked Thin-billed Prions were sampled over several seasons. Although individuals moulting in an area in one year were more likely to do so again in the subsequent year, several birds changed between Antarctic and South American moulting areas or vice versa [22].

Hence, both genetic and phenotypic mechanisms could cause a change in migratory direction if birds that migrated (and moulted) north suffered a higher mortality than birds migrating to the Antarctic. There is, in fact, evidence that Thin-billed prions were affected by several large-scale mortality events, with major wrecks in New Zealand in 1974 and 1986 [23], in South Africa in 1984 [24] and in Brazil in 1954, 1982, 1984 and 1996 [25]. During the 1996 event in Brazil alone over 10,000 individuals were beached, and unknown numbers of birds died at sea. These major wrecks all occurred in the middle of winter (July-August), beached birds were underweight, without large fat deposits and with empty stomachs and many birds that were found dying soon recovered once receiving adequate food. These observations suggest that winter mortality can be high over the Patagonian Shelf and other northern moulting areas, and that the most likely cause of these events are food scarcities in mid winter. Abnormal weather conditions were not regularly observed during these events, and were interpreted as proximal cause of strandings of birds in already poor condition [25]. The present data suggest that winter mortality might have hit preferentially northern moulting birds (Fig. 3), although data on mortality in the south are lacking.

Given their large populations there are surprisingly few sightings of Thin-billed prions in the Antarctic, and their winter distribution has mainly been inferred from beached recoveries. This may be partly due to the absence of observers in the vast open ocean area, and due to the fact that Thin-billed prions are not concentrated at the ice edge as some other, more readily observed species [26].

Thin-billed prions are not the only seabird migrating polewards for the winter. Blue petrels *Halobaena caerulea *are a closely related species, which replace Thin-billed prions in some parts of their circumpolar range, such as South Georgia. They have very similar stable isotope signatures [[27], adult Blue petrel feathers from South Georgia: δ^13^C = -24.1 ± 0.9 and δ^15^N = 7.9 ± 0.5], indicating similar latitude and trophic level during moult. In fact, they are often observed in feeding flocks together with prions [28].

## Conclusions

In conclusion, the present data strongly suggest a change of migration patterns in a small pelagic seabird. There are fundamental differences from the migration of land birds with their major flyways and overwhelmingly equator ward direction. In the polar oceans, the primary productivity is restructured in winter, and can be very high in near-surface waters, thus generating migration towards the polar areas. Our data from Thin-billed prions further support this view, and indicate that seabird migration patterns may quickly respond to changes such as those caused by global warming.

## Methods

The fieldwork was carried out in the framework of long-term studies and under licence (Falkland Islands Government Environmental Planning Department, Umweltbundesamt Germany).

### The Southern Ocean carbon isoscape

Authors sampling in different areas of the Southern Ocean noted a southward decline in δ^13^C of phytoplankton and subsequently, higher, trophic level organisms [9,10]. Across the Drake Passage, the area relevant to the present study, a 7‰ gradient in the δ^13^C of suspended particulate organic matter (POM) has been observed, from -23.2 ‰ at 53°S to values as low as -30.3 ‰ at 62°S [11].

The southward decline in δ^13^C does not track the abrupt changes in water chemistry and plankton species composition associated with the Polar Front Zone, but show a rather gradual change with latitude [11,29]. Studies did not report any significant changes in phytoplankton carbon or nitrogen concentrations or C/N ratios with latitude (e.g. Drake Passage: [11]], which would be indicative of differences in plankton standing crop or biochemistry (e.g., lipid content). The latitudinal change in δ^13^C was, however, highly correlated with sea surface temperatures and with the calculated concentration of CO_2_(aq) at equilibrium with atmospheric CO_2 _[11,29], suggesting that CO_2_(aq) significantly influences δ^13^C in ocean surface waters, and throughout the food chain.

**Figure 4:**
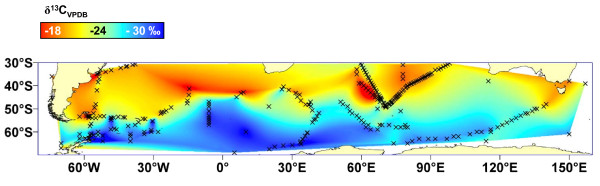
**The calculated isoscape after interpolation**. Sample locations are marked with crosses. All data were included as given in the original papers, for further details, please see the methods section.

For latitudinal trends and comparison of trophic levels (Fig. 1A), we compiled data published in the Southern Ocean for δ^13^C of phytoplankton and POM [29-41], planktonic crustaceans [10,12,33,36-40,42-48] and pelagic fish and squid [10,12,32,37,38,40,42,44,48-52]. Gerhard Fischer, University of Bremen, kindly provided an unpublished dataset from Fig. 1 of [34]. We used ArcView 9.3 (ESRI), to plot the distribution of 326 sampling locations for phytoplankton and POM from 30°S southwards (Fig. 4), and we used a nearest-neighbor interpolation in the Spatial Analyst tool to model and visualize the latitudinal trend in δ^13^C (Fig. S1, see also Fig. 1A,B).

### Study site, study species and feather samples

Samples of **Thin-billed prions **were collected as part of an ongoing study at New Island Nature Reserve, Falkland Islands (51°43'S, 61°17'W) during three consecutive breeding seasons (2003/04, 2004/05, 2005/06). We collected samples representing birds outside the breeding season (adult rectrices and undertail coverts), as well as the breeding season (chick undertail coverts), by gentle pulling and placed them in individual plastic bags. The first tail feathers of chicks including the tail coverts start to emerge after 3 weeks of age [[16], p. 427: 'at 22 d the sheaths of the rectrices can be felt protruding']; therefore, the distal parts of these feathers represent the middle of the nestling growth period of 50 d (i.e. about 25 d).

**Wilson's storm-petrels **have a circumpolar breeding distribution in the Antarctic. This species is described as a transequatorial migrant [28], although winter records in the southern hemisphere are also common, for example off Brazil in the Atlantic [18] or off Peru in the Pacific [28]. This northerly migration movement of Wilson's storm-petrels was well reflected by the stable isotope signatures of adult and chick feathers sampled at King George Island, South Shetlands [9,14], and stable isotopes helped to establish that these birds move further south, towards the sea ice edge, to feed up for egg production [9] and that their wintering areas differ between years, depending on the location of rich frontal systems [14]. Wilson's storm-petrels moult their feathers in the winter quarters, and thus, at a similar time to Thin-billed prions (see below).

Sample collection took place in the Tres Hermanos (Three Brothers Hill) colony on King George Island (62°14'S, 58°40'W), South Shetland Islands in the maritime Antarctic from December 1995 to April 2000. About 2000 pairs breed in this colony [53], and we have previously described their general breeding biology [summarized in [54]], diet [15] and provisioning patterns [[55] and references therein]. In 1995-1996, we collected 27 induced feathers of adult Wilson's storm-petrels as part of a ptilochronology study. The outermost right rectrix was pulled during a first control in February 1996 and induced feathers were collected at the time of recapture, in late March 1996. The time of feather growth was thus in the chick-rearing period, simultaneously with growing chick feathers.

### Comparison of stable isotope ratios in chick feathers and simultaneously grown induced adult feathers

The findings of a previous analysis indicated that adult and chick feathers undergo a similar isotopic enrichment compared to blood within and between species of Procellariiformes [56]. We compared 27 adult and 11 chick feathers of Wilson's storm-petrels grown simultaneously during the breeding season (Fig. 1C). They were not isotopically distinct (Wilk's λ = 0.96, P = 0.493). As wing, tail and body feathers are grown in the second part of the long nestling periods of petrel chicks, the tissue has already undergone a nearly complete tissue turnover since hatching. Although isotopic enrichment compared to diet was not determined specifically, similar isotope values in chicks and adults suggest that in these species, chick feathers reliably reflect the diet during the breeding season.

### Historical feathers

We included 115 feather samples from museum specimens of Thin-billed prions as listed below, including 82 samples from birds collected in the Falkland Islands and adjacent waters in the breeding season, and 33 samples of birds collected dead on beaches in Chile, Argentina and Brazil. We further included 14 historical feathers from Wilson's storm-petrels collected in the South Shetland Islands. Preferably, undertail feathers were taken, or flank feathers if sampling undertail feathers was not permitted. Sequentially moulted primaries indicated that most adults maintained highly conserved isotope values over the entire wing moulting period [22], and previous analyses had shown that undertail feathers, rectrices and flank feathers did not show significantly different stable isotope values. Because in Thin-billed prions, all moult occurs outside of the breeding season [16], and chicks of Thin-billed prions do not fledge until March, we are confident that flank feathers, rectrices and undertail feathers all represent the late autumn and early winter period (April-July). Tail feathers are moulted towards the end of the wing moulting period, which takes 2-4 months in prion species [57], and thus, tail feathers are moulted in early winter in Thin-billed prions.

We obtained samples of Thin-billed prions of the following museums specimens: American Museum of Natural History New York, USA: #445528-445541, 445544-445549, 445552-445564, 445566-445577, 445579-445594, 792675, 792883-792887, Natural History Museum, Tring, UK: # 1925.9.14.1, 1925.9.14.2, 1940.12.6.76, 1932.7.2.16, 1932.7.2.17, 1932.7.2.19-1932.7.2.23, 1949.52.10, 1951.51.2, 1969.2.2, 1969.2.2, 1972.14.1, Museo Argentino de Ciencias Naturales Bernardino Rivadavia, Buenos Aires, Argentina: #42973, 42974, 52110, 52111, 52331, 52372, 52406, 52414, 52770-52774, Naturhistorisches Museum Wien, Austria: NMW 80.541 - 80.550, NMW 87.177, Biozentrum Grindel und Zool. Museum Hamburg, Germany: # 75.106, 75.107, 75.133, Cornell University Museum of Vertebrates, USA: # 25006, 25007, National Museum of Natural History Leiden, Netherlands: # 3269, Museo de La Plata, Argentina: #13326, Museu de Ciências e Tecnologia - PUCRS, Porto Alegre, Brazil: # MCP0705, MCP1116, MCP1120, MCP1395.

We obtained samples of Wilson's storm-petrels of the following museums specimens: American Museum of Natural History New York, USA: # 196198, 196199, 196202, 349447, Natural History Museum, Tring, UK: # 1924.5.8.28, 1924.5.8.29, 1925.10.4.26, 1933.10.16.1, 1933.10.16.2, 1940.12.7.48, 1963.41.2-5.

### Sample preparation and stable isotope analysis

Carbon and nitrogen isotope analyses were carried out on 0.65-0.7 mg aliquots, weighed into tin cups. A single feather per sample was cut into small fragments using stainless steel scissors. Studies have shown that mean δ^13^C values did not shift when feathers were cleaned, supporting the notion that carbon stable isotope ratios are quite robust to minor contamination [58,59], and this is unlikely to be of any relevance given that differences observed in the present study between birds moulting in Antarctic and more northern areas were very large (-19 vs. -25‰). As cleaning agents may remove contaminants but may also change feather isotope values, either by leaving a residue with a different enough stable isotope ratio to change the measured value or by causing atom exchange [e.g. [59]], we decided not to apply a cleaning protocol. We thus selected feathers free from any obvious contamination, both in recent and historical feathers.

Carbon and nitrogen isotope ratios were measured simultaneously by continuous-flow isotope ratio mass spectrometry (CF-IRMS) using a Costech Elemental Analyser (EA) linked to a Thermo Finnigan Delta Plus XP Mass Spectrometer. Two laboratory standards were analysed for every 10 unknown samples, allowing any instrument drift over a typical 14 hour run to be corrected. Stable isotope ratios were expressed in δ notation as parts per thousand (‰) deviation from the international standards V-Pee dee belemnite (carbon) and AIR (nitrogen), according to the following equation δ X = [(R _sample_/R _standard_) - 1] × 1000 where X is ^15^N or ^13^C and R is the corresponding ratio ^15^N/^14^N or ^13^C/^12^C. Based on internal standards (tryptophan), the analytical precision (± 1 SD) was estimated as ± 0.18‰ and ± 0.17‰ for δ^15^N and δ^13^C, respectively.

### Data analysis

Data analysis was carried out using SPSS 11.0. The isotopic ratios of storm-petrel feathers grown simultaneously were compared between groups using discriminant analysis (Wilk's λ). We ran general linear models GLM with time and species as categorical independent variables ('factor'). As a measure of effect sizes we included partial eta-squared values (*η*^2^) i.e. the proportion of the effect+error variance that is attributable to the effect. The sums of the η^2 ^values are not additive [e.g. [60]]. Normality was tested by Kolmogorov-Smirnov-tests and visual inspection. Only prion winter data (adult feathers) were not normally distributed. This was due to outliers (5 of 62 data points), and we therefore followed the suggestion of [61] and considered the test results only significant when P < 0.01. Otherwise, significance was assumed at P < 0.05, and means are given with standard errors.

### Historical samples

Pools of fossil CO_2 _are depleted in ^13^C compared to CO_2 _in the atmosphere. The burning of large amounts of fossil CO_2 _has diluted the atmospheric CO_2 _pool resulting in a more negative δ^13^C value. This has been termed the Suess effect [7]. The increasing concentration of CO_2 _in the atmosphere has also resulted in an increasing concentration of dissolved CO_2 _in the ocean, resulting in increasing δ^13^C values in phytoplankton during the last 150 years. When long time series of δ^13^C are analysed, the data have to be corrected for the Suess effect [7]. We normalized all data to the current end of our database, the year 2008, by subtracting a year-specific factor δ^13^C = -1+ 1.1 ^(2008-year)*0.027 ^[7]. It should be noted that the effect is small compared to the large range of δ^13^C values observed here. For example, the estimated overall Suess effect from 1850 to 2002 is 0.62‰ for Falkland Island waters.

Rising sea surface temperatures reduce the amount of dissolved CO_2 _in the ocean, termed CO_2_(aq). Because δ^13^C in phytoplankton is negatively correlated with CO_2_(aq) [30], this leads to a minor enrichment of ^13^C, e.g. for the period 1850-2002 a maximum correction factor of 0.16‰ was modelled [7]. Because of this small influence, we did not correct for sea surface temperature variation.

## Competing interests

The authors declare that they have no competing interests.

## Authors' contributions

PQ, JFM, and RWF planned and carried out the study. RARMcG conducted the stable isotope analyses at the NERC Life Sciences Mass Spectrometry Facility, MA facilitated access to museums and hosted a Synthesys-funded museum study. PQ drafted the manuscript. All authors read and approved the final manuscript.
